# Low-dose aspirin in the prevention of pre-eclampsia in China (APPEC study): protocol for a multicentre randomized controlled trial

**DOI:** 10.1186/s13063-018-2970-3

**Published:** 2018-11-06

**Authors:** Li Lin, Yuchun Zhu, Boya Li, Huixia Yang, the APPEC Study Group, Xiaotian Li, Yangyu Zhao, Dunjin Chen, Hongbo Qi, Weishe Zhang, Hongjuan Ding, Meihua Zhang, Xianlan Zhao, Shihong Cui, Yang Mi, Xiaotong Sun, Yuyan Ma

**Affiliations:** 1 Department of Obstetrics and Gynaecology, Peking University First Hospital, No. 1 Xi’anmen Street, Xicheng District, Beijing, 100034 China; 2 Beijing Key Laboratory of Maternal Foetal Medicine of Gestational Diabetes Mellitus, Beijing, 100034 China

**Keywords:** China, Pre-eclampsia, Low-dose aspirin, Prevention, Pregnancy

## Abstract

**Background:**

Low-dose aspirin (LDA) has been proposed as a safe and inexpensive prophylactic agent. Studies in European/Western populations have shown promising results indicating that LDA can reduce the occurrence of pre-eclampsia (PE) in women with identifiable risk factors. However, few controlled trials, particularly large randomized controlled trials, have been performed in Asian populations. The aim of this project is to evaluate the effect of LDA for PE prevention on high-risk pregnant women in China, where PE is highly prevalent and the LDA supply status is commonly suboptimal.

**Methods/design:**

An open-label, multicentre randomized controlled trial is being conducted at 13 tertiary hospitals in 11 provinces in China. A total of 1000 eligible women with high-risk factors for developing PE according to their medical histories are being randomized into two groups: a control group (*n* = 500) and an intervention group (*n* = 500). Women with high-risk factors, such as a history of PE, chronic hypertension, type 1 or 2 diabetes, advanced maternal age, obesity, family history of PE or nulliparity are eligible. The control group is advised to undergo routine examinations, whereas the intervention group undergoes the routine examinations and receives LDA. LDA (100 mg/d) should be prescribed at night, initiating from early pregnancy (12–20 weeks of gestation) and lasting until 34 weeks of gestation. Demographic data and clinical endpoint outcomes, as well as biological samples (e.g., maternal blood, cord blood, amniotic fluid and placental samples), will be collected. The primary outcome is the occurrence of PE, and the secondary outcomes include maternal and neonatal outcomes and maternal biomarker levels.

**Discussion:**

This is the first and largest multicentre randomized controlled trial to assess the effect of LDA in preventing PE in a Chinese population. The results will potentially influence the prenatal care recommendations in China regarding intervention with LDA for PE.

**Trial registration:**

ClinicalTrials.gov, NCT02797249. Registered on 7 June 2016.

**Electronic supplementary material:**

The online version of this article (10.1186/s13063-018-2970-3) contains supplementary material, which is available to authorized users.

## Background

Pre-eclampsia (PE) affects approximately 3–5% of pregnant women [1], and this prevalence varies from country to country. It has been reported that the prevalence of PE is 4% in low- and middle-income countries [2] and 2.8% in China [3]. Although its prevalence is low, PE can cause substantial maternal and perinatal morbidity and mortality [4]. When left untreated, severe complications of PE, such as eclampsia, stroke, pulmonary oedema or kidney failure, can develop, and they can all be fatal [5]. Moreover, PE is also associated with preterm birth, foetal growth restriction and babies who are small for gestational age (SGA) [1]. Once PE develops, the only effective treatment is prompt delivery, with serious neonatal harm when far from term (< 34 weeks of gestation) [6]. Therefore, it is necessary to determine an effective early identification strategy and undertake a preventive measure for women at high risk of PE in order to reduce the prevalence of the complication.

For the early prevention of PE, the most consistent and promising prophylactic agent appears to be low-dose aspirin (LDA) [6]. Aspirin has become one of the most commonly used drugs, given its role as an analgesic, antipyretic and cardiovascular prophylactic agent. Studies have shown that aspirin can decrease the occurrence of pregnancy disorders, including PE [7], preterm birth and foetal growth restriction [8], on the basis of its anti-inflammatory and anti-thrombotic effects. One of the mechanisms by which aspirin exerts its beneficial effect is the selective inhibition of thromboxane synthesis without affecting prostacyclin synthesis, thus restoring a balance between thromboxane and prostacyclin [9, 10]. Besides, aspirin seems to improve defective trophoblast syncytialization by altering the production of specific cytokines, decreasing apoptosis and altering cell aggregation and fusion [11]. Moreover, in hypoxic conditions, aspirin can inhibit the expression of soluble fms-like tyrosine kinase 1 (sFlt1) in human trophoblasts and thus shows pro-angiogenic activity [12].

In most previous studies, the recommended dose of aspirin for PE prevention has been 60 to 150 mg/d, and 100 mg/d is used most commonly [13]. Studies recommend the initial use of aspirin after 12 weeks of gestation, and there is no research on aspirin treatment before 12 weeks of gestation [6]. Previous studies also have shown that the prevention of PE and foetal growth restriction using aspirin has a dose-response effect, and thus LDA is recommended to be initiated before 16 weeks of gestation [14]. Regarding drug safety, aspirin appears not to increase the rate of placental abruption, postpartum haemorrhage or foetal intracranial haemorrhage [6].

The prophylactic use of LDA for PE was first reported in 1978 [15], and since then numerous studies have reported the potential value of LDA in PE prevention among high-risk pregnant women. Authors of a Cochrane meta-analysis published in 2004 reported that the administration of antiplatelet agents in high-risk women could reduce the rate of PE by 19% [16]. The World Health Organization and several countries, including the United States, the United Kingdom, Canada and Australia, also recommended in their guidelines the use of LDA for the prevention of PE in high-risk pregnant women [17–21].

However, regarding the details of aspirin as a preventive treatment for PE, guidelines differ considerably from country to country. In addition, most randomized controlled trials (RCTs) have focused on Caucasian or black individuals; data for Asian populations are lacking [22]. Besides, only a few small-sample studies in China have been reported [23, 24]. There is scant evidence from large RCTs to guide the use of LDA for PE prevention for Chinese pregnant women. Therefore, we decided to conduct a large-population RCT in China to evaluate the role of LDA prophylactic treatment for PE prevention to fill this important gap in the literature and to ultimately translate research into practice by providing policy recommendations.

## Methods/design

### Study design

The low-dose Aspirin in the Prevention of Pre-eclampsia in China (APPEC) study is designed as multicentre, open-label RCT research. This study is being conducted at 13 tertiary hospitals in 11 provinces in China: Beijing, Henan, Guangdong, Shanghai, Jiangsu, Shandong, Hunan, Gansu, Shan-xi, Shanxi and Chongqing. A total of 1000 eligible women will be recruited and randomized into the control group or the intervention group, with an allocation ratio of 1:1. The start of the recruitment was in December 2016, and we anticipate that the study will be completed in approximately 3 years.

### Objective

The aim of this study is to evaluate the effect of LDA administered from early pregnancy in Chinese pregnant women with high-risk factors. The following hypothesis will be tested: The administration of LDA to pregnant women from early pregnancy (12 to 20 weeks of gestation) will reduce the occurrence of PE by 20%. Moreover, clinical information and maternal biological samples will also be collected for further mechanistic research.

### Participants

Participants will be eligible for registration in this study if they meet all of the inclusion criteria and none of the exclusion criteria, listed below.

#### Inclusion criteria

1. Aged ≥ 18 and < 55 years
2. Singleton pregnancy
3. Live foetus at gestational age 12–20 weeks
4. Be at high risk of developing PE based on clinical risk factors such as the following:
  a. At least one high-risk factor, namely history of PE, diabetes mellitus (type 1 or 2) or chronic hypertension
  b. At least two intermediate-risk factors, including obesity (≥28 kg/m^2^), advanced maternal age (≥ 35 years), family history of PE (mother and/or sister) or nulliparity
5. Fit to undergo all procedures listed in the protocol
6. Able to provide written informed consent

#### Exclusion criteria

1. Allergic to aspirin
2. Asthma
3. Peptic ulcers
4. Severe heart, liver or renal disease in which the patient cannot tolerate the treatment
5. Rheumatic immune disease
6. Mental disease
7. Alcohol or drug abuse
8. In vitro fertilization
9. Previous registration in another drug trial within the previous 3 months
10. Difficulty in undergoing any procedure listed in the protocol

### Randomization

Randomization will be performed using a web-based computerized central randomization system, the Interactive Web Response System (http://bjdx.clinicalcloud.com.cn/Login.aspx), in a 1:1 allocation ratio. The website randomly assigns a randomization code to each participant. Eligible women will be randomized to one of the two groups. Knowledge of the treatment allocation is open to the investigators and the participants.

### Sample size calculation

For the primary outcome indicator, “pre-eclampsia,” we assume that the estimated detection rate for PE is 20% in the control group, according to previous studies [25–27]. The occurrence of PE in the intervention group is supposed to be 16%, and thus the estimate of risk reduction with the intervention is 20%. For 80% power and a level of significance set at 0.05, 446 participants are required in each group. Thus, the total number of women required in the study is 892. Accounting for 10% withdrawal/loss to follow-up, 981 women will need to be recruited. Finally, we set the number of women to be recruited into the study at 1000 (500 per group). The power calculation was performed using PASS 2011 software (NCSS, Kaysville, UT, USA).

### Recruitment and trial procedure

Participants will be identified for their first antenatal care (before 20 weeks of gestation) in our study sites. The enrolment of patients will be at the obstetrics clinic, and a brief assessment card has been designed to determine the eligible women. Recruitment strategies are dependent on the local hospitals, including recruitment advertisement, online news, advertisements in training classes for pregnant women, and doctor screening. Our study sites can determine the most effective method for their hospitals.

**Fig. 1:**
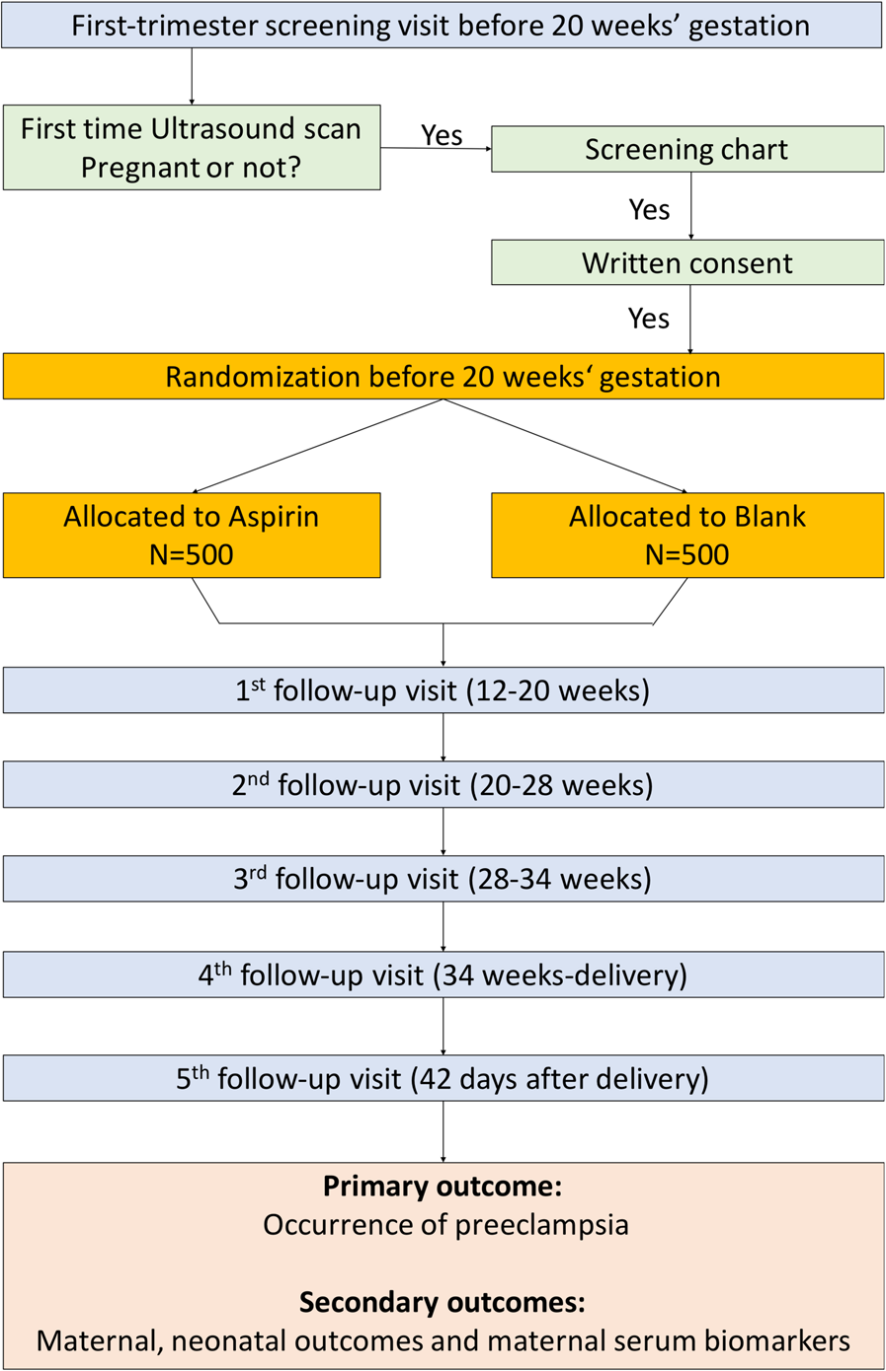
Flow chart of participants in the screening study and the randomized controlled trial

**Fig. 2:**
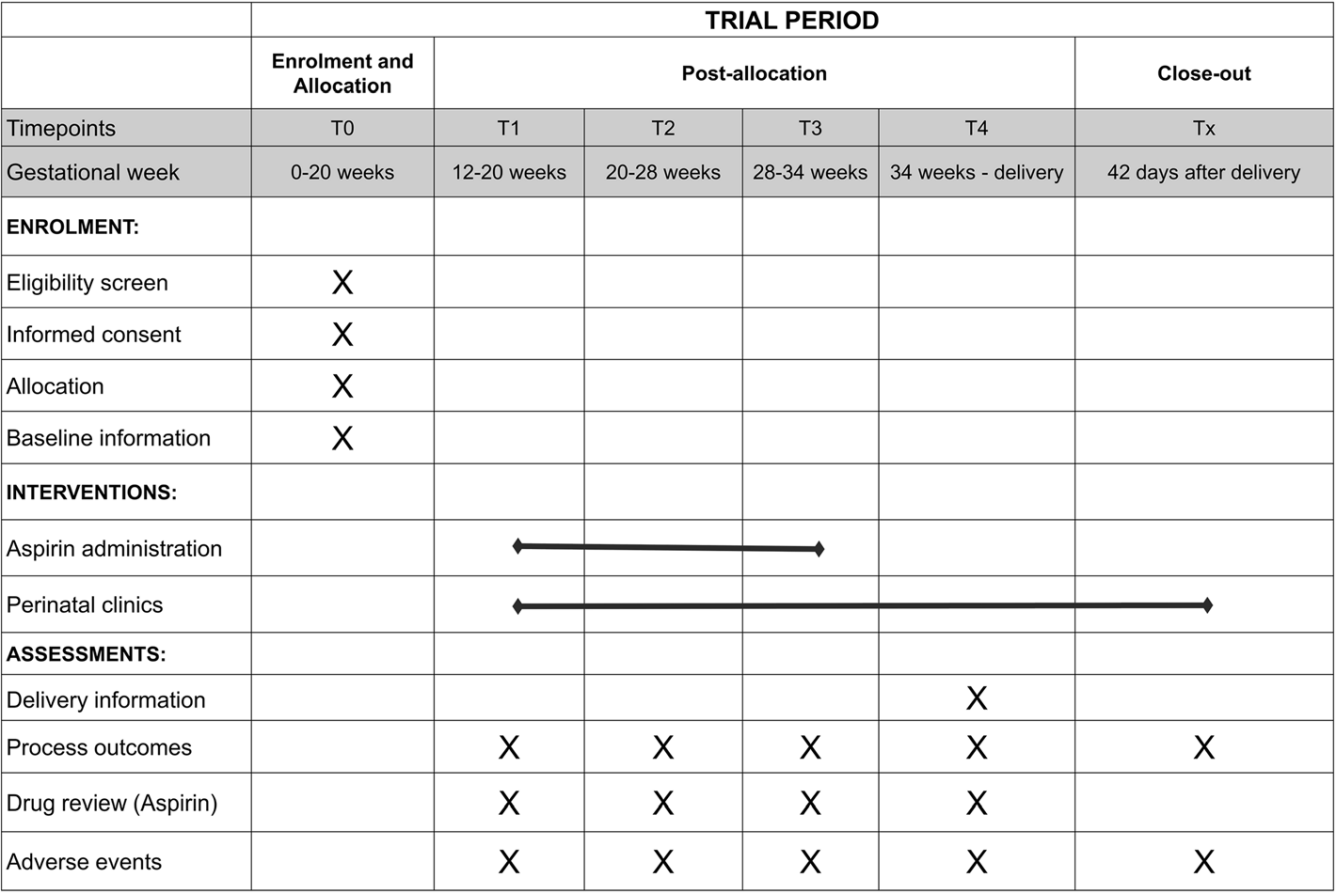
Standard Protocol Items: Recommendations for Interventional Trials (SPIRIT) figure. Schedule of enrolment, interventions and assessments for the APPEC (low-dose Aspirin in the Prevention of Pre-Eclampsia in China) study

Pregnancy is confirmed via transabdominal B-mode ultrasound. Then the investigators will evaluate the risk factors and baseline conditions of pregnant women, and those who meet the eligibility criteria will be enrolled in the study. Trial information will be provided verbally and with a written information sheet. Eligible women will be encouraged to take time to consider involvement in the study and to discuss the participation with their partners and/or family prior to providing written consent. All eligible women will provide written informed consent and receive written information about the trial drug. In addition, participants will be randomized and assigned with a randomization code, which will determine who receives 100 mg of aspirin daily and who does not. Details of antenatal and postpartum care are outlined in Figs. 1 and 2 and Additional file 1, which present the study flow chart; the Standard Protocol Items: Recommendations for Interventional Trials (SPIRIT) schedule of enrolment, interventions and assessments; and the SPIRIT checklist, respectively [28]. All of the participants will be free to withdraw from the study at any time if they wish, and appropriate on-going antenatal care will be arranged for the pregnant women according to their clinical risks.

### Intervention and control groups

Women will be assigned into one of two groups. The control group will receive standard antenatal care for high-risk women, whereas the intervention group will receive standard antenatal care for high-risk women plus aspirin prophylactic treatment. LDA (100 mg/d, enteric-coated aspirin tablets; Bayer Corporation, Leverkusen, Germany) should be taken nightly from the recruitment time (at 12–20 weeks of gestation) and stopped at 34 weeks of gestation or in the event of early delivery. This is an open-label RCT, and all of the participants, clinicians and investigators will be aware of the study group assignment.

### Specimen collection

Blood samples will be routinely collected at each visit. These samples will be stored and will be measured only after delivery. Cord blood and placental samples will be collected at delivery. Biomarkers (such as sFlt1, soluble endoglin [sEng] and placental growth factor [PlGF]) and further mechanistic research on PE and the effect of aspirin prevention will be performed. These results will not be made available to the managing clinicians and will not affect management.

### Study outcomes

#### Primary outcome

The primary outcome is the occurrence of PE. PE will be defined according to the American College of Obstetricians and Gynecologists 2013 recommendation [19]. The systolic blood pressure should be 140 mmHg or more, and/or the diastolic blood pressure should be 90 mmHg or more on at least two occasions 4 h apart, developing after 20 weeks of gestation in previously normotensive women (blood pressure < 140/90 mmHg). There should be proteinuria of 300 mg or more in 24 h, a urinary/protein creatinine ratio of at least 0.3 mg/dl or two readings of at least 2+ on dipstick analysis of midstream or catheter urine specimens if no 24-h collection is available. Efficacy will be assessed by the development of PE at any gestation after 20 weeks of pregnancy as defined above.

#### Secondary outcomes

The secondary outcomes include maternal and neonatal outcomes, maternal serum biomarkers (including sFlt1, sEng and PlGF) and placental samples. The key secondary outcomes are shown in Table 1.

**Table 1:** Key secondary outcomes of the APPEC study

Maternal	Preterm PE
	Severe PE
	Caesarean section delivery
	Placental abruption
	Antepartum haemorrhage
	Postpartum haemorrhage < 500 ml and < 1000 ml
Infant	Preterm birth
	Low birth weight
	Gestational age at delivery
	SGA < 10th percentile
	Intrauterine foetal death
	Stillbirth
	Neonatal intensive care unit admission
	Neonatal death
Placental and angiogenic growth factors	sFlt1
	sEng
	PlGF

*Abbreviations: APPEC* Low-dose Aspirin in the Prevention of Pre-Eclampsia in China, *PE* Pre-eclampsia, *PlGF* Placental growth factor, *sEng* Soluble endoglin, *sFlt1* Soluble fms-like tyrosine kinase 1, SGA Small for gestational age

### Data collection and analysis

#### Data collection

Data will mainly be collected prospectively in three rounds of surveys and from medical records. The baseline survey will be administered by a health worker at a local hospital in the first antenatal visit after obtaining written informed consent from women who have agreed to participate in the study. The follow-up surveys will be conducted at the hospital after within 2 days of delivery by a healthcare worker. The final survey will be a phone interview at day 42 after delivery. In addition, clinical data will be collected from the local hospital.

Participant data will be collected from a paper case report form (CRF) and then entered into an electronic case report form (eCRF) (http://bjdx.clinicalcloud.com.cn/Login.aspx) for further data sorting. Data on pregnancy and neonatal outcomes will be collected from the hospital maternity records or the general medical practitioners.

#### Data management

The paper CRFs are stored at the project office in Peking University First Hospital. The personal information of the participants will be accessible only to the data management staff; other study implementation team members, including data analysts, will not have access to it.

To ensure high-quality trial conduct, data management will be carried out according to the principles of the International Conference on Harmonization Good Clinical Practice Guideline. Quality control will be carried out routinely. Data types, entries and permitted ranges for answers to every question on the eCRFs are restricted on the web-based system being used. Automatic validation checks and automatic queries are raised by the system immediately to individual sites in the case of any query. Authorized individuals from the APPEC study group may also check the data for quality and may pose manual queries to the research site. The data will be stored by the APPEC study group for 20 years after the final publication of the trial. Further details on data storage, curation and destruction are available in a separate document on request to the APPEC trial unit.

### Statistical analysis

Baseline characteristics will be calculated using Student’s *t* test, the chi-square test or Fisher’s exact test as appropriate for comparisons across groups. Data will be analysed on an intention-to-treat basis to compare primary and secondary outcomes in both groups. The primary analysis will be done with generalized estimating equation regression models of the occurrence of PE, with adjustment for the effect of the risk factors for PE at screening and the study sites. The OR with 95% CI will be calculated to quantify the treatment effect. Moreover, exploratory subgroup analysis will be performed to identify whether the effects of intervention vary with the risk factors for PE, including chronic hypertension, pre-existing diabetes, history of PE, obesity, advanced age, nulliparity and family history of PE. The initial intervention weeks of gestation (e.g., < 16 weeks vs. ≥16 weeks of gestation) and its interaction with aspirin preventive treatment will also be investigated. A *P* value < 0.05 will be considered statistically significant. Statistical analysis will be performed by a statistician using IBM SPSS Statistics version 22.0 (IBM, Armonk, NY, USA) and Stata version 11.0 (StataCorp, College Station, TX, USA) software.

### Monitoring

#### Safety monitoring

Both adverse events (AEs) and serious adverse events (SAEs) will be prospectively recorded for all participants. Both will be recorded in terms of potential causality and severity. AEs will be recorded on the CRF. SAEs will be formally reported to the sponsor and principal investigator so that reasonable action can be taken. The potential causality of the AEs in relation to the experimental drug will be assessed and classified into six degrees (unrelated, unlikely to be related, possibly related, most likely related, definitely related and unable to assess), according to the guidelines of the Uppsala Monitoring Centre [29].

#### Potential complications/side effects

Data on AEs will be collected. All SAEs will be immediately reported to the project leader, who will be responsible for notifying the ethics committee, all participating investigators and the manufacturer of the study products.

## Discussion

The purpose of this APPEC study is to evaluate the effect of LDA on PE prevention in pregnant women with high-risk factors in China. As is known, PE is a serious life-threatening complication for both mother and foetus, especially in developing countries. However, there is no effective treatment for patients with PE, other than prompt delivery of the baby when severe PE develops. Therefore, an effective drug or treatment for PE would have a major beneficial impact for both maternal and perinatal health.

To our knowledge, this study is the first and largest RCT to determine the role of LDA in reducing the occurrence of PE in a Chinese population. We enrolled eligible women from 13 hospitals in 11 provinces in China, which may generally represent the whole population in China. From our study, we can learn whether routine administration of LDA is suitable for Chinese pregnant women with mixed high-risk factors. This study may influence future guidelines for the recommendation of LDA for Chinese pregnant women. Another strength of this study is that we will collect both clinical information and biological samples (serial serum samples and tissues). We will measure the circulating levels of biomarkers (such as sFlt1, sEng and PlGF) in the serial serum samples. These anti-angiogenic factors are considered to play important roles in inciting maternal endothelial dysfunction and the end-organ injury seen in PE. We will also focus on the novel mechanistic study of PE and aspirin’s preventive effect using these serial samples.

In conclusion, our study is novel in its assessment of the effect of LDA for PE prevention in a large Chinese population, which will be helpful in guiding healthcare providers regarding whether they should recommend LDA as a preventive drug to patients in China. Because there is no clear evidence that such agent is any more or less effective in reducing the relative risk for any particular subgroup, further research is needed to focus on the evaluation of LDA’s preventive effect in women with a single risk factor, such as nulliparity, chronic kidney disease, obesity and so forth. Besides, a recent study showed that aspirin’s effects on cardiovascular and oncological diseases varies with different body sizes and that a one-dose-fits-all approach to aspirin is unlikely to be optimal [30]. It is unknown whether the preventive effect of aspirin on pregnant women varies with their different body sizes. Therefore, this information should also be discussed with women at risk of PE to help them to choose a suitable dose of aspirin for PE prevention. Overall, this study will provide information for preventive treatment with aspirin in Chinese pregnant women, especially for populations with a high risk of PE.

### Trial status

The recruitment process was started from December 2016, and it is now recruiting participants. It is anticipated that the study will last for almost 3 years.

## Additional file

Additional file 1: SPIRIT 2013 checklist: Low-dose aspirin in the prevention of pre-eclampsia in China (APPEC study): protocol for a multicentre randomized controlled trial. (DOCX 52 kb)

## Abbreviations

AE: Adverse event
APPEC: Low-dose Aspirin in the Prevention of Pre-Eclampsia in China
CRF: Case report form
LDA: Low-dose aspirin
PE: Pre-eclampsia
PlGF: Placental growth factor
SAE: Serious adverse event
sEng: Soluble endoglin
sFlt1: Soluble fms-like tyrosine kinase 1
SGA: Small for gestational age
SPIRIT: Standard Protocol Items: Recommendations for Interventional Trials
